# The value of polyurethane-cuffed endotracheal tubes to reduce microaspiration and intubation-related pneumonia: a systematic review of laboratory and clinical studies

**DOI:** 10.1186/s13054-016-1380-8

**Published:** 2016-06-24

**Authors:** Stijn I. Blot, Jordi Rello, Despoina Koulenti

**Affiliations:** Department of Internal Medicine, Ghent University, Campus UZ Gent, De Pintelaan 185, 9000 Ghent, Belgium; CIBERES, Universitat Autonòma de Barcelona, Barcelona, Spain; Burns Trauma and Critical Care Research Centre, The University of Queensland, Brisbane, Australia; 2nd Critical Care Department, Attikon University Hospital, Athens, Greece

**Keywords:** Intubation, Endotracheal tube, Polyurethane cuff, Leakage, Secretions, Microaspiration, Ventilator-associated pneumonia, Pneumonia, Respiratory infection, Prevention

## Abstract

**Background:**

When conventional high-volume, low-pressure cuffs of endotracheal tubes (ETTs) are inflated, channel formation due to folds in the cuff wall can occur. These channels facilitate microaspiration of subglottic secretions, which is the main pathogenic mechanism leading to intubation-related pneumonia. Ultrathin polyurethane (PU)-cuffed ETTs are developed to minimize channel formation in the cuff wall and therefore the risk of microaspiration and respiratory infections.

**Methods:**

We systematically reviewed the available literature for laboratory and clinical studies comparing fluid leakage or microaspiration and/or rates of respiratory infections between ETTs with polyvinyl chloride (PVC) cuffs and ETTs with PU cuffs.

**Results:**

The literature search revealed nine in vitro experiments, one in vivo (animal) experiment, and five clinical studies. Among the 9 in vitro studies, 10 types of PU-cuffed ETTs were compared with 17 types of PVC-cuffed tubes, accounting for 67 vs. 108 experiments with 36 PU-cuffed tubes and 42 PVC-cuffed tubes, respectively. Among the clinical studies, three randomized controlled trials (RCTs) were identified that involved 708 patients. In this review, we provide evidence that PU cuffs protect more efficiently than PVC cuffs against fluid leakage or microaspiration. All studies with leakage and/or microaspiration as the primary outcome demonstrated significantly less leakage (eight in vitro and two clinical studies) or at least a tendency toward more efficient sealing (one in vivo animal experiment). In particular, high-risk patients intubated for shorter periods may benefit from the more effective sealing capacity afforded by PU cuffs. For example, cardiac surgery patients experienced a lower risk of early postoperative pneumonia in one RCT. The evidence that PU-cuffed tubes prevent ventilator-associated pneumonia (VAP) is less robust, probably because microaspiration is postponed rather than eliminated. One RCT demonstrated no difference in VAP risk between patients intubated with either PU-cuffed or PVC-cuffed tubes, and one before-after trial demonstrated a favorable reduction in VAP rates following the introduction of PU-cuffed tubes.

**Conclusions:**

Current evidence can support the use of PU-cuffed ETTs in high-risk surgical patients, while there is only very limited evidence that PU cuffs prevent pneumonia in patients ventilated for prolonged periods.

**Electronic supplementary material:**

The online version of this article (doi:10.1186/s13054-016-1380-8) contains supplementary material, which is available to authorized users.

## Background

Intubation-related pneumonia, including ventilator-associated pneumonia (VAP) or early postoperative pneumonia, remains a feared complication in the acute healthcare setting. Despite a plethora of preventive measures and growing attention to care bundle initiatives for patients at risk, the average occurrence rate of VAP among intensive care unit (ICU) patients intubated for at least 2 days ranges from 10 % to 20 % [1–5]. This is particularly worrisome, given the substantial clinical and economic burden of this infectious complication [6]. With an excess length of ICU stay of 5–7 days, there exists an important cost-saving potential [3].

The main pathogenic mechanism of intubation-related pneumonia is bacterial translocation from the stomach and/or oropharynx to the lower respiratory tract. Within a few hours following endotracheal intubation, pathogenic microorganisms colonize the oropharyngeal mucosa, dental plaque, stomach, and sinuses. As such, microbiologically contaminated subglottic secretions accumulate above the cuff of the endotracheal tube (ETT). While the purpose of the cuff is to seal the extraluminal airway, its effectiveness is not flawless. When conventional high-volume, low-pressure (HVLP) cuffs are inflated, channel formation due to folds in the cuff wall can occur. These channels facilitate microaspiration of subglottic secretions, even when the cuff pressure (P_cuff_) is adequate (20–30 cmH_2_O) [7].

Ultrathin polyurethane (PU) cuffs have been developed to minimize the channel size within the folds of an inflated cuff. While the cuff membrane thickness of conventional polyvinyl chloride (PVC) cuffs is about 50–70 μm, the cuff wall thickness of PU cuffs ranges from 7 to 10 μm [8]. Therefore, the intention of these PU-cuffed ETTs is that the relatively smaller cuff channels will minimize microaspiration of subglottic secretions and thus the risk for intubation-related pneumonia. The purpose of this study was to systematically review the evidence, including that from laboratory and clinical investigations, concerning the effectiveness of PU-cuffed ETTs to minimize fluid leakage (microaspiration) and intubation-related pneumonia compared with conventional PVC-cuffed ETTs.

## Methods

### Search strategy

The PubMed, EMBASE, and Web of Science databases were each systematically searched using the following key terms: “endotracheal” OR “tracheal,” “poly-urethane” OR “polyurethane,” AND “cuff.” Additionally, the snowball method was used to detect potentially eligible studies. The literature searches were performed on 7 December 2015 without time or language restriction. No predefined review protocol was registered.

### Study selection

Eligible studies included nonclinically (i.e*.*, laboratory) or clinically controlled studies in which fluid leakage (either laboratory or clinical studies) or intubation-related pneumonia (clinical studies) was compared between PU- and PVC-cuffed ETTs. Search results were screened by title and abstract. Selected papers underwent a full-text assessment, and eligibility issues were resolved between authors. Reference inclusion and exclusion criteria for this review are described in Table 1.

**Table 1:** Literature selection criteria

Inclusion criteria
• Experimental and clinical studies
• Intervention arm with high-volume, low-pressure (HVLP) polyurethane (PU)-cuffed endotracheal tube
• Control group with HVLP conventional polyvinyl chloride (PVC)-cuffed endotracheal tube
• Studies evaluating either fluid leakage or intubation-related respiratory infection (early-postoperative pneumonia, ventilator-associated tracheobronchitis, ventilator-associated pneumonia)
• Article written in English or Dutch
Exclusion criteria
• Descriptive studies
• Studies without a control group
• Evidence of confounders in one of the study arms, such as the use of additional measures to reduce the risk of microaspiration (e.g., subglottic secretions drainage, taper-shaped cuff, gel lubrication of the cuff, positive end-expiratory pressure) or use of measures to prevent pneumonia (e.g., hand hygiene promotion, head-of-bed elevation) in only one of the study arms. Differences in cuff shape other than conical (taper-shaped) were not considered an exclusion criterion. As such, comparisons of cylindrical PU cuff vs. globular PVC cuffs were accepted.
• Not original research (reviews, systematic reviews, meta-analysis, editorials, letters)
• No full text available

### Data extraction and quality assessment

Extracted data included study setting, design, and sample size; implemented interventions; definitions; presence or volume of fluid leakage; and rates of respiratory infections. The methodological quality (i.e., risk of bias) of included randomized controlled trials (RCTs) was assessed using the Jadad quality scale, which assigns a score ranging from 0 to 5 points, with a score ≥3 indicating a high-quality study [9]. Evaluation criteria in the Jadad scale assessment include randomization, blinding, and accounting for all study subjects. For non-RCTs, the risk of bias was assessed using the Downs and Black tool [10]. This score is based on 27 questions related to reporting of the data, internal and external validity, and factors potentially confounding internal validity. Summaries of the methodological evaluations of included RCTs and non-RCTs are shown in Additional file 1.

According to the objective of this review, for studies evaluating multiple types of ETTs, only comparisons of PU-cuffed ETTs with conventional PVC-cuffed tubes were selected (see also Table 1 for inclusion and exclusion criteria). If necessary, statistical analyses were performed on reported data in the original articles (Fisher’s exact test for comparison of leakage and/or pneumonia rates). Where necessary, the authors of the original studies were contacted to provide additional information.

## Results and discussion

**Fig. 1:**
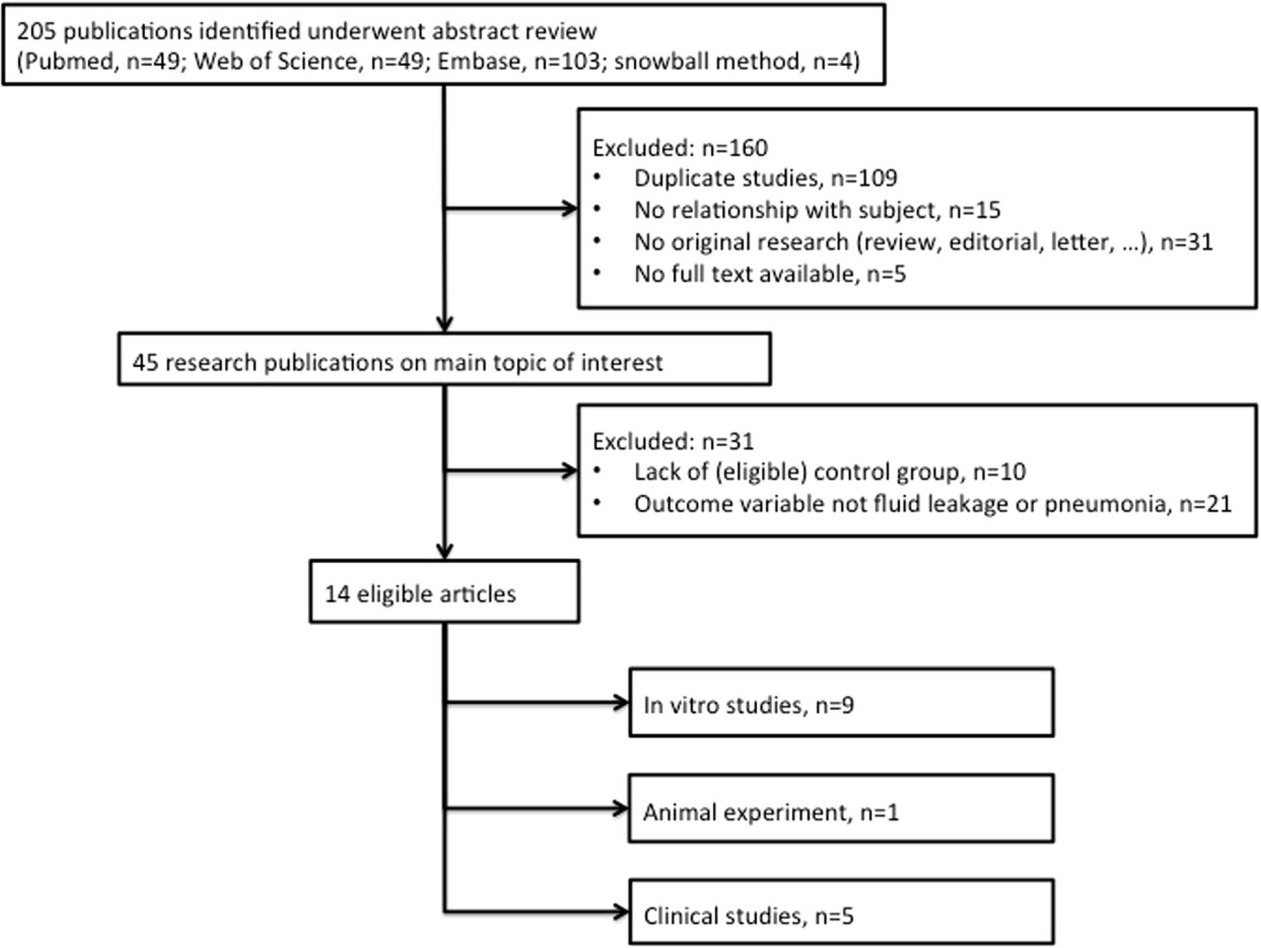
Flowchart of the literature search

The search strategy yielded 205 records. Following title, abstract, and full-text assessments, 14 publications were included for final evaluation (Fig. 1). The studies included nine different in vitro experiments (Table 2), one in vivo experiment (Table 2), and five clinical studies (Table 3).

**Table 2:** Summary of laboratory studies

Author, year	Materials tested	Experimental settings	P_cuff_	Fluid and leakage measurement	Experiment repetition	Main results
In vitro studies						
Dullenkopf, 2003 [8]	• 1 PU-cuffed ETT (MICROCUFF HVLP ICU; Microcuff GmbH, Heidelberg, Germany) • 4 PVC-cuffed ETTs (Portex Profile Soft Seal, SIMS Portex Ltd., Hythe, UK; Rüschelit Super Safety Clear, Rüsch GmbH, Kernen, Germany; Mallinckrodt Hi-Lo, Mallinckrodt Medical, Athlone, Ireland; Sheridan/CF, Hudson Respiratory Care, Temecula, USA) • ID all ETTs 7.5 mm	• Vertical PVC trachea model (ID 20 mm) • Unlubricated and lubricated cuffs • No ventilatory simulation	• 10, 15, 20, 25, 30, 60 cmH_2_O • Controlled by manometer	• Colored water (5 ml) • Amount of leaked fluid passed through tube cuff within 5, 10, and 60 minutes	4 repetitions of 2 tubes of each brand	• Fluid leakage occurred in all PVC cuffs at P_cuff_ 10–60 cmH_2_O. In the PU cuff, leakage was recorded only at P_cuff_ 10 cmH_2_O (<5 minutes) and at P_cuff_ 15 cmH_2_O (<60 minutes) • PU cuffs sealed significantly better at conventional P_cuff_ (20–30 cmH_2_O) than with all types of PVC cuffs; while no leak was observed in PU cuffs, >90 % of the test fluid (>4.5 ml) passed through cuff in all PVC cuffs
Lucangelo, 2008 [14]	• PU-cuffed ETT (Mallinckrodt SealGuard, Mallinckrodt Medical, Cornamady, Anthlone County, Ireland) • PVC-cuffed ETT (Mallinckrodt Hi-Lo, Mallinckrodt Medical, Cornamady, Anthione County, Ireland) • ID all ETTs 7.5 mm	• Room temperature • Vertical Pyrex cylindertrachea model, ID 25 mm • Unlubricated cuffs • 5-, 7.5-, and 10-cm PEP randomly applied • PEP removed after 30 minutes • Experiment lasted 1 h	• 30 cmH_2_O • Controlled by aneroid manometer	• Evans blue (1 ml) diluted with normal saline (1 ml)	Triplicate experiments with new ETTs	• 10 minutes after PEP removal (40 minutes after Evans blue dye application on top of cuffs), all liquid had passed through PVC cuffs • No leakage in PU cuffs
Dave, 2010 [24]	• PU-cuffed ETTs (standard SealGuard, Covidien, Athlone, Ireland; MICROCUFF, Kimberley Clark, Zaventem, Belgium) • 3 PVC-cuffed ETT (Hi-Lo, Covidien, Athlone, Ireland; Portex Profile Soft Seal, SIMS Portex Ltd., Hythe, UK; Rüschelit Super Safety Clear, Rüsch GmbH, Kernen, Germany) ID all ETTs 7.5 mm	• Room temperature • Artificial PVC trachea model with variable ID 16, 20, 22 mm • Unlubricated cuffs • No ventilatory simulation, no PEEP	• 25 cmH_2_O • Controlled by digital automated manometer	• Clear water (5 ml) • Leakage measured at 5 minutes and 60 minutes	8 tubes tested of each ETT type	PVC cuffs leaked considerably more than PU cuffs • At 5 minutes: - ID 16 and 20 mm: 0.03–1.62 ml (PU) vs. 4.27–4.67 ml (PVC) - ID 22 mm: 0–4.85 ml (PU) vs. 4.65–4.71 ml (PVC) • At 60 minutes: - ID 16 and 20 mm: 0.90–4.58 ml (PU) vs. 4.61–4.69 ml (PVC) - ID 22 mm: 0–4.85 ml (PU) vs. 4.65–4.75 ml (PVC)
Dave, 2011 [13]	• PU-cuffed ETT (SealGuard, Covidien, Athlone, Ireland) • PVC-cuffed ETT (Hi-Lo, Covidien, Athlone, Ireland) ID all ETTs 5 mm	• Vertical artificial PVC trachea model, ID 22 mm • Unlubricated cuffs • Test lung attached to trachea model • Pressure-controlled ventilation with PIP 15, 20, 25 cmH_2_O • PEEP 5 and 10 cmH_2_O • Closed tracheal suction system attached with variable suction pressure (-20000 or -30000 Pa) during 5, 10, 15, and 20 seconds	• 25 and 50 cmH_2_O • Controlled by digital automated manometer	• Clear water (10 ml)	4 tubes tested of each ETT type	PU cuffs leaked less (range 0–0.12 ml) than PVC cuffs (range 0.05–6.28 ml) at all P_cuff_ levels (*p* < 0.001) and at all negative suction pressures tested (*p* < 0.001)
Kolobow, 2011 [27]	• 2 PU-cuffed ETTs (prototype tube with Lycra PU cuff, MICROCUFF, Kimberly-Clark, Kimberly-Clark Health Care, Roswell, Georgia, USA) • PVC-cuffed ETT (Hi-Lo, Mallinckrodt/Covidien, Boulder, Colorado, USA) ID all ETTs 8 mm	• Vertical acrylic tube trachea model, ID 20 mm • Cuffs not lubricated • No ventilatory simulation, no PEEP	• 20 cmH_2_O • Controlled by aneroid manometer	• Methylene blue-colored water (15 ml) • Leak time observation 24 h • Leakage expressed as milliliters per hour	6 tubes tested of each ETT type	• No leakage in Lycra PU prototype ETT • The other PU cuff (MICROCUFF) allowed less leakage (1.2 ± 0.4 ml/h) than PVC cuff (1.2 ± 1.3 ml/h) (*p* < 0.001)
Ouanes, 2011 [25]	• PU-cuffed ETTs (MICROCUFF, Kimberly-Clark, Zaventem, Belgium) • PVC-cuffed ETT (Rüschelit Super Safety Murphy, Rüsch GmbH, Kernen, Germany) ID ETTs 7.5 and 8 mm	• Vertical artificial Plexiglas trachea model, ID 18 mm • Cuffs not lubricated • Ventilatory settings 10 cmH_2_O PSV driven by active inspiration with trigger sensitivity at 2 L/minute and respiratory rate at 20 breaths/minute. Three effort intensities (occlusion pressure at 0.1 second): low = 2 cmH_2_O, moderate = 5 cmH_2_O, and high = 10 cmH_2_O • Experiments performed with PEEP 0 and 5 cmH_2_O	• 30 cmH_2_O • Controlled by manual manometer every 3 h	• Methylene blue (1 ml) diluted with 4 ml normal saline • Leak time observation 1 h • Leakage expressed as milliliters per hour	3 tubes tested of each ETT type	At all inspiratory levels tested, significantly less leakage occurred with PU cuffs: mean 0.5 (SD 0.5) ml/h vs. 36.8 (SD 31.6) ml/h (*p* < 0.001)
Zanella, 2011 [15]	• PU-cuffed ETTs (MICROCUFF, Kimberly-Clark, GA, USA) • 3 PVC-cuffed ETT (Mallinckrodt Hi-Lo, Mallinckrodt, NY, USA; Mallinckrodt High-Contour, Kimberly-Clark, Zaventem, Belgium; Portex Ivory, Smiths Medical, UK) ID all ETTs 8 mm	• Vertical PVC cylinder as trachea model, ID 20 mm • Cuffs not lubricated • Ventilatory settings: CPAP simulation with 4 levels of PEEP randomly applied (0, 5, 10, and 15 cmH_2_O)	• 30 cmH_2_O • Continuously controlled by water seal valve providing continuous positive pressure into pilot balloon	• Methylene blue-dyed water added above the cuff to make water column 10 cm • Leak time camera observation 24 h with picture taken every 60 seconds • Leakage expressed as water column above cuff (cm) • Leakage detected by blinded observer	5 tubes tested of each ETT type, at PEEP 5–15 cmH_2_O 5 new ETTs tested at PEEP 0	Leakage observed after 24 h: • PU cuff: - No leakage at PEEP levels 5, 10, and 15 cmH_2_O - Minor leak without PEEP (about 2 cm) • 3 types of PVC cuffs: -No leak at PEEP 15 cmH_2_O - Minor leak at PEEP 10 cmH_2_O (about 2 cm) - Major leak at PEEP 5 cmH2O (>8 cm) - Maximum leak without PEEP (10 cm)
Li Bassi, 2013 [11]	• 8 HVLP ETTs with various cuff characteristics, among which were: PU-cuffed ETT (Kimvent MICROCUFF, Kimberly-Clark Health Care, Roswell, GA, USA) PVC-cuffed ETTs (Rüschelit Safety Clear Plus, Teleflex, Limerick, PA, USA; Hi-Lo, Covidien-Nellcore and Puritan Bennett, Boulder, CO, USA) • ID 7, 7.5, 8 mm	• Artificial trachea model (ID 18, 20, and 22 mm) oriented 30 degrees above horizontal • Cuffs not lubricated • No ventilatory simulation • Short-term fluid sealing capacity (1 h): 3 ETT IDs at 4 P_cuff_ levels • Long-term fluid sealing capacity (24 h) with 4 best-performing ETTs at 1 h; experiment performed with 7.5 mm ID ETTs and P_cuff_ 30 cmH_2_O • Measurement: multivariate analysis in which leakage rate was evaluated considering several other cuff characteristics: P_cuff_, cuff OD, cuff length, PU cuff, taper-shaped cuff, ratio cuff OD/trachea model ID, ratio cuff OD/cuff length, cuff compliance, and trachea model ID	• 15, 20, 25, 30 cmH_2_O • Controlled by automated manometer	• Oropharyngeal secretion simulant (viscosity 3 cP at shear rate 75/second) • Leakage recorded as milliliters per hour • Leak time observation 1 h and 24 h	3 tubes tested of each ETT type	PU cuffs leaked substantially less than PVC cuffs: • Short-term mean fluid leakage rates (1 h): - PU cuff (MICROCUFF): 0.09 ± 0.06 ml/h - PVC cuff (Rüschelit): 4.46 ± 3.47 ml/h - PVC cuff (Mallinckrodt Hi-Lo): 2.24 ± 1.93 ml/h • Long-term mean fluid leakage rates (24 h): - PU cuff (MICROCUFF): 0.69 ± 0.36 ml/h - PVC cuff (Mallinckrodt Hi-Lo): 114.74 ± 16.79 ml/h • In multivariate analysis (considering 8 types of ETTs with various cuff characteristics), internal P_cuff_, cuff OD, and cuff length were independently associated with lower leakage rates; cuff material did not reach statistical significance in this model
Lau, 2014 [23]	• PU-cuffed ETTs (Kimvent MICROCUFF, Kimberly-Clark Health Care, US) • PVC-cuffed ETT (Portex, Smiths Medical International Ltd., UK) ID all ETTs 8 mm	• Silicone cylinder trachea model (ID 20 mm) oriented 35 degrees above horizontal • Cuffs not lubricated • Ventilatory settings: 5 simulated mechanical ventilation scenarios, including different PEEP levels and disconnection with and without spontaneous breathing effort. Each scenario was tested under 3 P_cuff_ levels and then repeated with application of continuous suction force (−200 cmH_2_O for 3 minutes)	• 10, 20, 30 cmH_2_O • Maintained by automated device	• Clear water (20 ml) • Leakage observation time 20 minutes. • Leakage was video-recorded and expressed as cumulative amount of leakage (ml)	2 ETTs of each type, and each ETT tested 4 times (8 measurements for each ETT per scenario and P_cuff_)	PU cuffs consistently demonstrated best protection against fluid leakage; clinical situations associated with greater leakage were mechanical ventilation without PEEP, circuit disconnection with spontaneous breathing, application of suction, and low P_cuff_ (10 cmH_2_O)
In vivo study						
Li Bassi, 2015 [26]	• PU-cuffed ETTs (KimVent MICROCUFF, Halyard Health, USA) • PVC-cuffed ETT (Rüschelit Safety Clear Plus, Teleflex Incorporated, Limerick, PA, USA) ID ETTs not reported	• Large White-Landrace pigs (37.3 ± 3.6 kg) randomized to be intubated with either of the test ETTs • 5 pigs in PU group • 4 pigs in PVC group • Intubated, ventilated, and anesthetized for 76 h • After 52 and 73 h, pigs were placed in prone, bed oriented 30 degrees above horizontal, and PEEP reduced to 0	• 28 cmH_2_O • Maintained by automated device	• Methylene blue (2 ml) and phosphate-buffered solution (3 ml) with 1.5 μl of 2.0-μm Invitrogen fluorescent microspheres (Life Technologies, Carlsbad, CA, USA) • 1 h from instillation, leakage estimated by presence of methylene blue and quantification of microspheres in tracheal secretions quantified by calculating percentage of recovered microspheres per gram of tracheal secretions per total amount of instilled microspheres	1 ETT per animal	• Methylene blue was never found in tracheal secretions • Percentage of aspirated microspheres was not significantly lower with PU cuffs (0.06 ± 0.05 %) than with PVC cuffs (0.12 ± 0.06 %)

*Abbreviations: ICU* intensive care unit, *PEP* positive expiratory pressure, *PSV* pressure support ventilation, *PU* polyurethane, *PVC* polyvinyl chloride, *ETT* endotracheal tube, *HVLP* high volume-low pressure, *ID* internal diameter, *P*_*cuff*_ cuff pressure, *PEEP* positive end-expiratory pressure, *PIP* peak inspiratory pressure, *PEP* positive expiratory pressure, *CPAP* continuous positive airway pressure, *cP* centipoise, *OD* outer diameter

Study arms or aspects of the experimental setting that have no relationship with the outcome of interest of this review are not included in the summary of the individual studies

**Table 3:** Summary of clinical studies

Author, year	Materials tested	Study design	P_cuff_	Outcomes	Main results
Lucangelo, 2008 [14]	• PU-cuffed ETT, cylindrical shape (Mallinckrodt SealGuard, Mallinckrodt Medical, Cornamady, Anthlone County, Ireland) • PVC-cuffed ETT, spindle-like shape (Mallinckrodt Hi-Lo, Mallinckrodt Medical, Cornamady, Anthlone County, Ireland)	• RCT • 2 groups of 20 ICU patients requiring immediate orotracheal intubation and mechanical ventilation because of deterioration of consciousness (GCS score ≤8) • Ventilatory settings: VC ventilation with V_T_ 8–9 ml/kg and respiratory rate to maintain normocapnia, and PEEP (5 cmH_2_O) • 5 h postintubation, PEEP was removed • Oral and tracheal secretions were not aspirated during experiment	• 30 cmH_2_O • Controlled with aneroid manometer	• Evans blue (1 ml) diluted in normal saline (1 ml) • Bronchoscopic evaluation to detect blue dye in trachea at 1 h and 5 h postintubation (with PEEP) and hourly thereafter until 12 h postintubation (without PEEP) • As soon as blue spot was seen on trachea caudal to ETT tip, experiment was finished	• 5 h postintubation, leakage observed in 2 patients in PVC group • At 6th hour (1 h after PEEP removal), leakage observed in all patients in PVC group • In the PU group, first leakage occurred after 8 h; at end of experiment (12th hour), fluid leakage absent in 3 patients • Difference between the 2 groups (log-rank test on Kaplan-Meier survival curves) was statistically significant (*p* < 0.001)
Poelaert, 2008 [30]	• PU-cuffed ETT, cylindrical shape (SealGuard, Covidien, Mansfield, Mass, USA) • PVC-cuffed ETT, cylindrical shape (standard Mallinckrodt, Mallinckrodt Inc. Hazelwood, Mo, USA) • Female patients: ID 8 mm • Male patients: ID 9 mm	• RCT, single-blind • 2 groups of 67 patients scheduled for cardiac surgery • Intraoperative antibiotic prophylaxis with cefazolin 2 g 3 times daily for 24 h	• 20–26 cmH_2_O • Controlled immediately after intubation, at closure of sternum, on arrival at ICU, and every 4 h during postoperative course	• Early postoperative pneumonia (until 7 days postoperatively) • Nosocomial pneumonia was defined as all of the following: - New/evolving infiltrate on chest x-ray - Temperature > 38.2 °C - Leukocytosis (>12000 cells/mm^3^) - Presence of purulent sputum/endotracheal aspirate - Increase in C-reactive protein for 2 consecutive postoperative days - Deterioration in PaO_2_/FiO_2_ ratio ≥20 % • Diagnosis: assessor blinded	• Rate of postoperative pneumonia in PU group significantly lower than PVC group (23 % vs. 42 %; *p* = 0.026) • In multivariate regression analysis, use of PU-cuffed ETTs appeared to be protective for early postoperative pneumonia (OR 0.31, 95 % CI 0.13–0.77)
Nseir, 2010 [40]	• PU-cuffed ETT, cylindrical shape (MICROCUFF, Kimberly-Clark, Zaventem, Belgium) • PVC-cuffed ETT, cylindrical shape (Mallinckrodt Hi-Lo Lanz, Mallinckrodt Medical, Athlone, Ireland)	• Prospective, observational trial in ICU patients • PVC group (patients included in first 6 months of study, *n* = 26); PVC group (patients included in second period of 6 months, *n* = 22) • Patients observed during 24 h with continuous monitoring of P_cuff_ • After 24 h, tracheal suctioning to obtain aspirate sample for pepsin measurement • Pepsin levels considered positive at 200 ng/ml	• 25 cmH_2_O • Manually adjusted every 8 h	• Pepsin in tracheal secretions (ng/ml) used as proxy for microaspiration of gastric contents • Recorded 24 h postintubation	• No difference in P_cuff_ variation observed between groups • Pepsin levels lower among patients with PU-cuffed ETTs (217 ± 126 ng/ml) than in PVC group (408 ± 282 ng/ml) • Pepsin levels >200 ng/ml more common in PVC group (69 % vs. 27 %; *p* = 0.008) • Pepsin levels >300 ng/ml more common in PVC group (61 % vs. 22 %; *p* = 0.009)
Miller, 2011 [32]	• PU-cuffed ETT, cylindrical shape (MICROCUFF, Kimberly-Clark Corporation, Roswell, GA, USA) • PVC-cuffed ETT, cylindrical shape (conventional type)	• Before-after study with interrupted time-series analysis • 1 hospital, 5 ICUs • Retrospective comparison of VAP rates in respective periods • 1 year of observation with PVC-cuffed tubes before intervention • 1 year of observation with PU-cuffed ETTs • 3 months postintervention observation (return to conventional PVC-cuffed ETTs)	Not reported	• VAP rates expressed per 1000 ventilation days • VAP diagnosed on basis of clinical or microbiologic criteria, based on CDC’s National Healthcare Safety Network standard definition [29]	• Baseline year of observation (PVC-cuffed ETT): 37 VAP episodes (5.3/1000 ventilation days) • Intervention year (PU-cuffed ETT): 21 VAP episodes (2.8/1000 ventilation days) (*p* = 0.0138) • After return to PVC-cuffed ETTs, 6 episodes in 3 months (3.5/1000 ventilation days) • Incidence risk ratio of VAP during intervention year 0.57 (95 % CI 0.34–0.96)
Philippart, 2015 [31]	• PU-cuffed ETT, cylindrical shape (MICROCUFF, Kimberly-Clark, Irving, Tx, USA) • PVC-cuffed ETT, cylindrical shape (Hi-Lo, Covidien, Dublin, Ireland) • PU-cuffed ETT, conical shape (SealGuard, Covidien, Dublin, Ireland) • PVC-cuffed ETT, conical shape (TaperGuard, Covidien, Dublin, Ireland) • ID 7.5 or 8 mm	• Multicenter RCT, 4 study arms • PU cylindrical group (*n* = 123) • PVC cylindrical group (*n* = 129) • PU conical group (*n* = 153) • PVC conical group (*n* = 129) At study inclusion, no important differences between groups were observed	• 25–30 cmH_2_O • Manually controlled with manometer every 6 h • Airway management standardized across study sites	• Primary endpoint: bacterial colonization of the trachea (10^3^ CFU/ml) at days 1, 2, 3, 7 • Secondary endpoint: cumulative VAP rate during ICU stay, defined on basis of clinical, biological and radiological patterns [41]; bacterial cultures sampled in all patients suspected of having VAP and confirmed if quantitative culture was at least 10^4^ CFU/ml	• No differences in tracheal colonization (at >10^3^, >10^4^, >10^5^, or >10^6^ CFU/ml) were observed 48 h postintubation (*p* > 0.05, *p* value indicates difference across 4 groups) • VAP rate between PU cylindrical and PVC cylindrical group not different (resp. 17.1 % vs. 10.8 %; *p* = 0.202) • VAP rate between PU conical and PVC conical group not different (respectively 16.3 % vs. 13.2 %; *p* = 0.505) • No difference in VAP rate observed when both PU groups and both PVC groups were pooled (respectively 16.6 % vs. 12.0 %; *p* = 0.140)

*Abbreviations: PU* polyurethane, *PVC* polyvinyl chloride, *RCT* randomized controlled trial, *GCS* Glasgow Coma Scale, *VC* volume-controlled, *V*_*T*_ tidal volume, *PEEP* positive end-expiratory pressure, *ID* internal diameter, *VAP* ventilator-associated pneumonia, *CDC* Centers for Disease Control and Prevention, *PaO*_*2*_ */FiO*_*2*_ ratio of arterial oxygen partial pressure to fractional inspired oxygen

Study arms or other aspects of the study that have no relationship with the outcome of interest of this review are not included in the summary of the individual studies

### In vitro studies

Among the 9 in vitro studies, 10 types of PU-cuffed ETTs were compared with 17 types of PVC-cuffed tubes, accounting for 67 vs. 108 experiments with 36 PU-cuffed tubes and 42 PVC-cuffed tubes, respectively (Table 2). The results of these studies were generally in favor of PU-cuffed tubes, primarily because they demonstrated no or, compared with PVC-cuffed tubes, less fluid leakage. Several aspects of the experimental setup should be considered, including shape and elasticity of the trachea model, positioning of the tube, room temperature, gel lubrication of the cuff, P_cuff_, viscosity of the fluid, ventilatory simulation and positive end-expiratory pressure (PEEP), additional cuff characteristics, and duration of the leakage observation. These aspects are summarized in Table 2.

Gel lubrication of ETT cuffs reduces the risk of microaspiration by plugging channels formed in the cuff wall. In one study, the sealing capacity of cuffs was tested with and without gel lubrication. Improved sealing was observed in all tested tubes except one PVC-cuffed tube [8]. On the one hand, an argument in favor of not using lubricated cuffs is that in such experiments the sole potential of cuff material to avoid leakage was evaluated. On the other hand, gel lubrication is standard in daily practice, albeit to smoothen the intubation maneuver in the first place.

The viscosity of the test fluid is another issue in which in vitro experiments might deviate from the clinical situation. All but one of the included in vitro studies used (dyed) water to evaluate the sealing ability of the cuffs. Li Bassi et al., however, used an oropharyngeal secretion simulant [11]. In this investigation, PU-cuffed tubes sealed more efficiently than PVC-cuffed tubes.

PEEP plays an important role in the risk of microaspiration because the upward pressure that it exerts on an ETT cuff enables better sealing capacities and thus reduces microaspiration and pneumonia [12]. Dave et al. [13] tested cuffed ETTs under various levels of positive pressure ventilation, with either 5 or 10 cmH_2_O PEEP, simulation of an endotracheal suction maneuver using a closed suction catheter with variable suction pressure (either −20000 or −30000 Pa), and variable duration of suctioning (5–20 seconds). Significantly less fluid leakage was observed among PU-cuffed tubes than in PVC-cuffed tubes, regardless of PEEP or suctioning settings. Lucangelo et al*.* demonstrated that PVC-cuffed ETTs leaked whether exposed to PEEP or not, while PU-cuffed ETTs did not leak under either circumstance [14]. Zanella et al. evaluated fluid leakage according to various PEEP levels (0, 5, 10, and 15 cmH_2_O) [15]. The PU-cuffed tubes demonstrated minor leakage in the absence of PEEP, while PVC-cuffed tubes leaked at all PEEP levels except 15 cmH_2_O. Lau et al. demonstrated that PU cuffs sealed better than PVC cuffs when PEEP levels were varied, and the ventilator was disconnected [8].

The target P_cuff_ for HVLP cuffs is 20–30 cmH_2_O, and all studies used a P_cuff_ within this range. In clinical practice, however, and in the absence of an automated P_cuff_ controller, the P_cuff_ is extremely variable due to some technical conditions (positive pressure ventilation, N_2_O ventilation, sedation, and neuromuscular blocking agents) [16–18], environmental circumstances (high altitudes, such as during helicopter transport) [19], and patient-related factors (e.g., core temperature, changes in patient positioning) [20–22]. For that reason, with some in vitro experiments, researchers additionally evaluated fluid leakage in case of low (<20 cmH_2_O) and high (>30 cmH_2_O) P_cuff_ [8, 11, 13, 23]. Overall, a higher P_cuff_ results in better sealing efficacy. Li Bassi et al. controlled for various cuff characteristics by means of multivariate analysis [11]. A greater cuff outer diameter was protective against fluid leakage in multivariate analysis, together with P_cuff_ and cuff length. Of note, cuff material had no significant impact in multivariate analysis, but it displayed a clear advantage in favor of PU cuffs in univariate analyses.

A caution pertaining to the amount of time allotted to observe leakage past the cuffs is warranted when interpreting the results of the in vitro experiments. The observation periods varied from 20 minutes [23], to 1 h [8, 14, 24, 25], and to 24 h [15, 26, 27]. Although these studies included observation periods that were sufficient to demonstrate significant differences between PU- and PVC-cuffed ETTs, it is critical to recognize that aspiration may occur in patients who are ventilated longer than 24 h. Indeed, exposure time is pivotal in device-associated infections such as VAP [28, 29].

### In vivo study

Li Bassi et al. conducted an investigation that included 29 pigs in which 7 different ETTs were tested [26]. Fluid leakage, as indicated by methylene blue or fluorescence microsphere detection, was compared between PU- and PVC-cuffed ETTs residing in five and four pigs, respectively. Leakage of methylene blue was not observed past either cuff type, but the percentage of microspheres per gram of tracheal secretion was lower in animals with PU cuffs than in those with PVC cuffs. The lack of statistically significant differences in leakage between the cuffs may be attributable to the limited number of pigs (i.e., power) that were compared.

### Clinical studies

Table 3 summarizes the clinical studies that compare PU- and PVC-cuffed ETTs. Three RCTs were identified that involved 708 patients, comprising 363 patients with PU-cuffed tubes and 345 with PVC-cuffed tubes [14, 30, 31]. The Jadad score indicated two high-quality trials [14, 30] and one trial with higher risk of bias [31] (Additional file 1). Lucangelo et al. measured microaspiration (leakage) of blue dye over 12 h, as evidenced by bronchoscopic evaluation [14]. One hour after PEEP removal, leakage became evident in all patients intubated with PVC-cuffed ETTs, but leakage was not observed in the PU group. However, at the end of the observation period, 17 of 20 patients in the PU group experienced microaspiration. This study demonstrates that, after PEEP is removed, microaspiration is relatively delayed with use of PU-cuffed tubes compared with its onset when ETTs with PVC cuffs are used. The clinical relevance of this observation is particularly pertinent to patients undergoing short-term intubation (e.g., surgical patients).

In the remaining two RCTs, the rate of early postoperative pneumonia and VAP were the respective outcomes of interest. Philippart et al. evaluated the risk of VAP among patients intubated with either PU-cuffed or PVC-cuffed ETTs [31]. The study included four different arms: a cylindrical PU group, a conical PU group, a cylindrical PVC group, and a conical PVC group. No differences in VAP rates were observed among the groups. Despite its multicenter and randomized design, however, that study had limitations. First, the study was not blinded. Second, randomization was not organized on an individual basis. Instead, the investigators predetermined clusters of nine or ten consecutive patients stratified by study centers, with each cluster being assigned to one of the study groups. It was anticipated that inclusion in a single cluster would cover a period of 4–6 weeks for which an appropriate number of ETTs were made available. This approach does not completely rule out bias generated by natural fluctuations in local epidemiology of nosocomial infection (e.g., outbreaks). Third, throughout the study period, P_cuff_ was controlled by a manual manometer every 6 h. As already mentioned, P_cuff_ is easily affected by several conditions and can as such be considered an uncontrolled factor potentially affecting the study results.

Two nonrandomized clinical studies with low risk of bias according to the Downs and Black tool [10] were identified (Additional file 1). Nseir et al. prospectively measured pepsin levels in tracheal aspirates to assess microaspiration in 48 patients. Despite the relative small study size, significantly less microaspiration was observed among patients intubated with PU-cuffed tubes. Of note, the observation period was limited to 24 h, thereby suggesting a potential advantage for short-term intubated patients, whereas the clinical benefit for patients with prolonged ventilation remained uncertain. This hypothesis is supported by data reported by Poelaert et al. [30]. In their high-risk cardiac surgical patients, the rate of early postoperative pneumonia was significantly lower among patients intubated with PU-cuffed tubes (23 % vs. 42 %; *p* = 0.026). In that study, the average periods of intubation and mechanical ventilation were 25 h and 19 h for patients in the PVC cuff and PU cuff groups, respectively (*p* = 0.22). In a post hoc analysis, Poelaert et al*.* determined that use of PU-cuffed ETTs in high-risk cardiac surgery patients is protective against early postoperative pneumonia in patients who are intubated for up to 16.6 h [29].

In another non-RCT, Miller et al. evaluated VAP rates in five ICUs during two periods of 1 year (baseline and intervention). In the baseline and intervention periods, respectively, PVC- and PU-cuffed ETTs were used. Thereafter, ICUs used PVC-cuffed ETTs and VAP rates were monitored for a 3-month period. The authors showed a favorable reduction in VAP rates following the introduction of the PU-cuffed ETT; however, nonrandomized studies are prone to bias, such as natural trends in local epidemiology of microbial ecology and nosocomial infection [32]. In addition, a Hawthorne effect cannot be excluded. The Hawthorne effect might have led to improved adherence in universal precautions in infection prevention (e.g., hand hygiene) or in more specific measures to avoid VAP (e.g., oral care), albeit that no changes in infection prevention policy were advocated during the study period.

The potential positive effect of a medical device to prevent microaspiration might be completely eliminated by the failure to control for adequate P_cuff_. No cuff seals efficiently in the absence of a sufficiently inflated cuff. At least three RCTs demonstrated that automated P_cuff_ controllers are effective in keeping P_cuff_ levels within the target range [33–35]. Therefore, it seems that automated P_cuff_ control is indispensable, regardless of the cuff material. In addition, the value of automated P_cuff_ controllers to avoid VAP was demonstrated in a recent meta-analysis [36].

While PU cuffs do not totally succeed in avoiding microaspiration, their advantage might strengthen other features of ETTs to avoid leakage. Lorente et al. reported the results of an RCT in which PU-cuffed tubes with subglottic secretion drainage (SSD) were compared with PVC-cuffed tubes without SSD [37]. The trial demonstrated significantly reduced rates of early and late VAP in the group of patients intubated with the PU-cuffed tube (+SSD).

### Limitations and future directions for research

This review has limitations. First, none of the clinical studies addressed safety issues. While there are no particular concerns linked with the use of PU-cuffed tubes, considering long-term safety data may be a point of interest in future studies. Second, the fact that tube characteristics other than cuff material may play a role in sealing efficacy is a limitation of this review. We could take into account neither differences in cuff length nor discreet differences in shape. Regarding the latter, we excluded comparisons of PU vs. PVC cuffs when cuff shapes were tapered and/or conical vs. cylindrical and/or globular in shape, as there are in vitro and clinical data demonstrating improved sealing capacity with taper-shaped cuffs [24, 38]. Finally, among the included studies, there appeared to be substantial differences in study designs, experimental setups, and endpoints. As such, it appeared very difficult to make a pooled estimate of the collected scientific evidence.

More clinical studies on the topic are welcome. RCTs with optimal control for confounding factors should be performed to further assess the value of PU-cuffed ETTs in the prevention of VAP. Special emphasis should be placed on identifying patients who might benefit the most from PU-cuffed ETTs to reduce VAP risk. Concerning early postoperative pneumonia, it may be worthwhile to focus on oncology patients, such as those with esophageal cancer undergoing esophagectomy, as these patients have a particularly high risk for developing postoperative pneumonia [39].

## Conclusions

The in vitro, in vivo, and clinical studies reviewed here provide evidence that, compared with PVC-cuffed tubes, PU-cuffed tubes protect more efficiently against microaspiration, or at least postpone substantial leakage of secretions. Indeed, all studies reviewed here that had leakage and/or microaspiration as the primary outcome demonstrated significant results [8, 11, 13–15, 23–25, 27, 40] or at least a tendency toward more efficient sealing [26] in association with PU cuffs. It should be mentioned that only two (single-center) clinical studies assessed microaspiration, including only one RCT. In particular, high-risk patients intubated for shorter periods seemed to benefit from the more effective sealing capacity of PU cuffs, as such patients experienced a lower risk of early postoperative pneumonia [30]. The evidence that PU-cuffed ETTs prevent VAP is less robust, because microaspiration is probably postponed rather than totally eliminated with use of PU cuffs. As such, current evidence can support the use of PU-cuffed ETTs in high-risk surgical patients, while there is currently only very limited evidence that PU cuffs prevent pneumonia in patients ventilated for prolonged periods.

## Abbreviations

CDC, Centers for Disease Control and Prevention; cP, centipoise; CPAP, continuous positive airway pressure; ETT, endotracheal tube; GCS, Glasgow Coma Scale; HVLP, high volume, low pressure; ICU, intensive care unit; ID, internal diameter; OD, outer diameter; PaO_2_/FiO_2_, ratio of arterial oxygen partial pressure to fractional inspired oxygen; P_cuff_, cuff pressure; PEEP, positive end-expiratory pressure; PEP, positive expiratory pressure; PIP, peak inspiratory pressure; PSV, pressure support ventilation; PU, polyurethane; PVC, polyvinyl chloride; RCT, randomized controlled trial; SSD, subglottic secretion drainage; VAP, ventilator-associated pneumonia; VC, volume-controlled; V_T_, tidal volume

## Additional file

Additional file 1: Online Supplementary Material 1. Risk of bias assessment of randomized clinical trials using the Jadad quality scale. Online Supplementary Material 2. Risk of bias assessment of non-randomized clinical studies (Downs and Black tool [10]). (DOCX 82 kb)
